# Identifying the Association between Surface Heterogeneity and Electrochemical Properties in Graphite

**DOI:** 10.3390/nano11071813

**Published:** 2021-07-13

**Authors:** Jaewon Kim, Alan Jiwan Yun, Kyeu Yoon Sheem, Byungwoo Park

**Affiliations:** 1 Department of Materials Science and Engineering, Research Institute of Advanced Materials, Seoul National University, Seoul 08826, Korea; yureru@snu.ac.kr (J.K.); hangyeolee@snu.ac.kr (A.J.Y.); 2 Samsung SDI, 130 Samsung-ro, Yeongtong-gu, Suwon 16678, Korea

**Keywords:** adsorption-energy distribution, fast-charging, cyclability, commercialized graphite

## Abstract

Graphite materials for commercial Li-ion batteries usually undergo special treatment to control specific parameters such as particle size, shape, and surface area to have desirable electrochemical properties. Graphite surfaces can be classified into basal and edge planes in the aspect of the structure of carbons, with the existing defect sites such as functional groups and dislocations. The solid-electrolyte interphase (SEI) mostly forms at the edge plane and defect sites, as Li-ions only intercalate through these non-basal planes, whereas the electrochemical properties of graphite largely depend on its surface heterogeneity due to the difference of reactivity on each plane. In order to quantify the detailed surface structure of graphite materials, local-absorption isotherms were utilized, and the analyzed nanostructural parameters of various commercial graphite samples were correlated with the electrochemical properties of each graphite anode. Thereby, we have confirmed that the fraction of non-basal plane and fast-charging capability has strong linear relations. The pore/non-basal sites are also related to the cycle life by affecting the SEI formation, and the determination of surface heterogeneity and pores of graphite materials can provide powerful parameters that imply the electrochemical performances of commercial graphite.

## 1. Introduction

Graphite is an excellent anode material for Li-ion batteries in terms of energy density, due to its low operating voltage (~0.1 V), acceptable theoretical capacity (372 mAh g^−1^), high electrical conductivity, and relatively low volume expansion (~13%) during lithiation/delithiation [1,2,3]. Most of all, low cost makes graphite more attractive as an anode material for current and post Li-ion batteries than other candidates (Si, Sn-alloying type, Li_4_Ti_5_O_12_, Li metal, etc.) [4,5,6,7,8,9,10,11]. On the other hand, because of the anisotropy and kinetic properties of flakes, graphite is used after some post treatment to achieve the desired electrochemical properties rather than using the raw material [12,13,14].

Due to the growing demand for electric vehicles and high energy storage systems, implementing rate capability and stable cycle performance of graphite has become more important. According to various fundamental and experimental studies, exposure of edge planes to electrolytes can be advantageous in terms of fast-charging characteristics, as diffusion of Li-ion is 4 orders of magnitude faster than that across the basal plane [15,16,17,18]. Also, many groups have confirmed that the cycle life of graphite electrode is majorly affected by the solid-electrolyte interphase (SEI) formation on the graphite surface, as a thin and dense SEI layer can suppress additional side reactions [19,20,21]. Our group also confirmed that the exposed pores in graphite, which can act as sites to promote SEI formation, are defective planes that reduce the cyclability of the graphite anode [22]. As stated above, it is clear that the surface and particle conditions of the graphite is closely related to the cycle performance and rate capability [23,24,25,26]. However, as the reactions on graphite anodes are kinetically different from the actual site on the surface, it is not easy to predict the actual performance with the surface characteristics of graphite materials. In order to correlate the complex relations of graphite surfaces and electrochemical performances more clearly, surface characteristics of graphite are required with more details.

To study the underlying relation between the surface and electrochemical performances, the characteristics of exposed surface planes should be characterized, in addition to the actual surface area. To determine the surface area and pore structure of graphite, it is typical to obtain a Brunauer–Emmett–Teller (BET) specific surface area through nitrogen-adsorption analysis [27]. However, the BET specific surface area is different from the actual graphite surface area because of the assumption that different graphite surfaces have the same physisorption energies [28,29,30]. Therefore, it is necessary to obtain specific surface areas with distinguishable edge, basal, and defect sites. To this end, the adsorption-energy distribution (AED) model can be utilized to classify the physisorption of gas on heterogeneous surfaces, based on the study of Ross and Olivier [31]. The basal plane, having a higher areal carbon density, will adsorb nitrogen more strongly than the less dense edge plane. In contrast, lattice defects in the graphene layers enhance the adsorbent–adsorptive interactions and lead to higher adsorptive energy [32,33,34].

Along with the above method, we have conducted nanostructural analysis to characterize various commercial graphite materials to examine the relation between graphite characteristics and electrochemical properties. First, the surface heterogeneity of each graphite sample is classified via analyzing the adsorption-potential energy. The tendency of the electrochemical performances was examined by comparing the fast-charging characteristics and lithium-ion kinetic properties through the non-basal plane (edge and defect sites). Various commercial artificial and natural graphite exhibited predictable linear relations with the fast-charging capability and cyclability in the full-cell battery.

## 2. Materials and Methods

### 2.1. Preparation of Graphite Samples

Various graphites with different surface properties were used in this work. The commercial artificial graphites were labelled after their electrochemical performances as follows: FG (fast-charging) and QG (fast-charging with high specific capacity) artificial samples, and AG (artificial graphite) and CG (good cyclability artificial) samples were prepared (particle size *D*50 ≅ 10 μm). For natural graphites (particle size *D*50 ≅ 17 μm), NG (natural spherical graphite) and TPP (triphenylphosphine-treated NG) were used [22]. All graphites used in this work were supplied by Samsung SDI Co., Republic of Korea.

### 2.2. Materials Characterization

The measurement of the volumetric nitrogen adsorption isotherms was conducted by Micrometrics ASAP (Micrometrics, Nacross, GA, USA) up to 1 bar at 77 K. Brunauer–Emmet–Teller (BET) methods and Barre–Joyner–Halenda (BJH) methods were performed to analyze the distributions of surface areas and pore size of samples, respectively. Adsorptive-potential distribution was calculated from the adsorption isotherms utilizing the software from Micrometrics, based on the DFT model. Samples were characterized using X-ray diffraction (D8 Advance: Bruker, Billerica, MA, USA) and field-emission scanning electron microscopy (SIGMA: Carl Zeiss, Germany) to analyze the crystal structures and morphologies, respectively. Chemical properties on the sample surfaces were analyzed via Raman spectroscopy (LabRAM HR Evolution: Horiba, Japan) with a 532-nm laser. Particle size distribution of the samples was measured by laser diffraction (HELOS (H3173) & RODOS: Sympatec GmbH, Germany).

### 2.3. Electrochemical Measurements

The graphite electrodes were fabricated using graphite as active materials, and styrene-butadiene rubber (SBR) and sodium carboxymethyl cellulose (CMC) were used as binders (weight ratio of 96.5:1.5:2.0). The binders were provided by Samsung SDI Co., Republic of Korea. The mixed slurry was deposited on the Cu foil by the doctor-blade method, and calendared by roll press, followed by the drying step at 110 °C in a vacuum overnight. The mass loading for the electrode was 14 mg cm^−2^, and the electrode was prepared to be thick enough (thickness of 98 μm) to have the electrode density of 1.55 g cm^−3^. For the electrolyte, 1.15 M LiPF_6_ in ethylene carbonate (EC)/dimethyl carbonate (DMC)/ethyl methyl carbonate (EMC) (volume ratio of 2:4:4, Panax Etec, Republic of Korea) was used with the addition of 1.5 wt.% of vinylene carbonate (VC). The cells were assembled in an Ar-filled glove box using CR2032.

WBCS3000S (WonATech Co., Republic of Korea) was used for the charge/discharge test of half-cells between 0.01 V and 1.5 V. In order to assemble the full cell, LiNi_0.88_Co_0.10_Al_0.02_O_2_ (NCA) was applied as a cathode material, and the capacity ratio of negative to positive electrodes (*N*/*P* ratio) was fixed to 1.1. The mass loading of NCA electrode was 22 mg cm^−2^, and its practical capacity was 200 mAh g^−1^. Electrochemical performance of the full cell was tested at the rate of 1 C (= 4.3 mA cm^−1^) for the first two cycles to form an SEI layer, and the cycle life was assessed for 300 cycles at 0.2 C between 2.5–4.2 V (CC–CV mode).

The cyclic voltammetry (CV) and electrochemical impedance spectroscopy (EIS) were conducted with the potentiostat (ZIVE MP1: WonATech Co., Republic of Korea). For the fabrication of the symmetric cells (graphite/graphite), two graphite electrodes with the state of charge (SOC) of 0% and 50% were employed, where both are collected from the identical half cells (graphite/Li). The EIS measurements for the symmetric cells were performed at the open-circuit voltage (OCV). For coin-type half cells, the measurement was carried out at 0.1 V, and the frequency range of the AC perturbation was 100 kHz to 10 mHz with an amplitude of 10 mV.

## 3. Results and Discussion

### 3.1. Heterogeneity Classification of Graphite Surface

**Figure 1:**
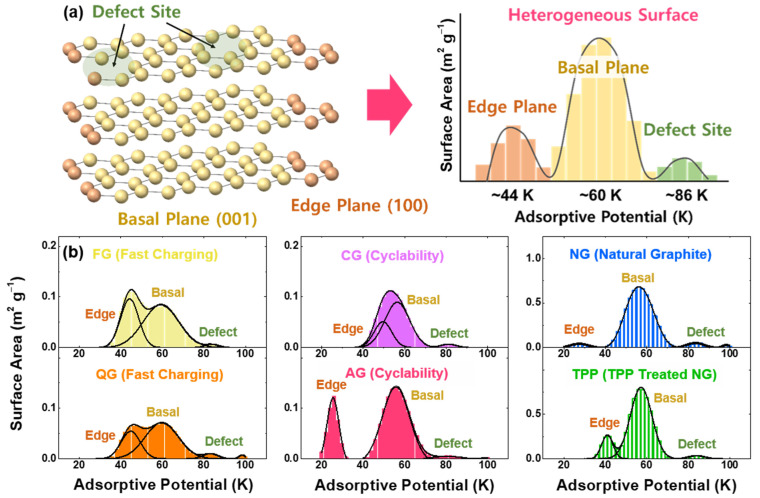
N_2_ adsorption on different kinds of graphite surfaces. (**a**) Schematics for the adsorption-potential distribution of heterogeneous graphite surfaces. (**b**) Surface area as evaluated by the adsorption energy. The characteristics of four artificial-graphite and two natural-graphite powders are as follows: FG (fast-charging), QG (fast-charging with high specific capacity), CG (cyclability), AG (artificial graphite), NG (natural spherical graphite), and TPP (triphenylphosphine-treated NG).

Graphite lattices can be divided into two planes, a basal and an edge plane. In addition, the surface of a graphite lattice is defective, as point defects, surface steps and functional groups exist. Along with the adsorption-energy distribution (AED) of the graphite [28,29,30,31], values of the specific surface areas can be obtained (Figure 1a). The adsorption-potential energy of nitrogen has been obtained from the van der Waals force model, with the potential region at approximately 60 K for the basal plane, ~26 and ~44 K for the edge plane, and ~86 and 96 K for the defect site (by *k_B_ T* where *k_B_* = 8.617 × 10^−5^ eV K^−1^) [32,33,34,35,36].

The representative adsorptive potential distributions of six commercial graphite samples with Lorentzian fitting are displayed in Figure 1b, with the detailed description of samples in the experimental part. Among the artificial graphite, FG (38% for the fraction of the non-basal plane, as shown in Table S1) and QG (34%) samples have excellent fast-charging capability, compared to AG (25%) and CG (29%) samples with good cyclability. For natural graphite, not only natural spherical graphite (NG) but also triphenylphosphine-coated NG (TPP) were used with an expectation that the phosphorus treatment can modify the ratio of basal/non-basal planes and reduce nanopores on the graphite surface. Notably, TPP (17%) has a larger non-basal plane than the NG (8%) sample. Apparent particle size distributions are shown in Figure S1.

**Figure 2:**
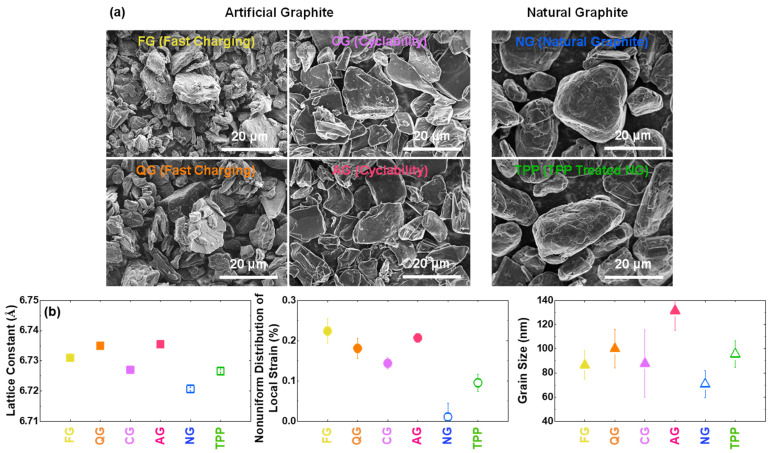
Structural characterization of graphite samples. (**a**) SEM images of graphite powders. (**b**) Lattice constant, non-uniform distribution of local strain, and grain size along the [001] direction, from the X-ray diffraction analysis.

SEM images in Figure 2a exhibit that artificial graphites have flake shapes, and natural graphite samples are potato-like, similar to the typical commercial spherical graphites. The sample with triphenylphosphine (TPP) shows no significant morphological changes from SEM. XRD analysis confirms that artificial graphites have larger lattice constants in the *c*-axis direction than natural graphite samples (Figure 2b), similar to those reported in other studies [37,38,39]. The TPP treated sample shows a slight increase in the lattice constant, probably due to phosphorus [22]. The nonuniform distribution of local strains of artificial graphites is higher than that of the natural graphites, which is considered to be an effect generated during the graphitization process [40,41]. However, the grain sizes in the *c*-axis direction are quite similar among the six graphite samples. Compared to the XRD analysis, which identifies all the structural states of graphite (with the X-ray effective depth of ~20 μm), Raman analysis further shows some structural changes of the graphite surface (in the range of ~100-nm depth) (Figure S2).

### 3.2. Correlation between the Fraction of Non-Basal Plane and Fast-charging Capability

In order to design a high C-rate electrode, it is well known that overall properties of active materials (particle size, shape, active area, etc.) and electrodes (tortuosity, loading level of active materials, etc.) need to be carefully optimized [23,24,42,43,44]. In addition, the overall rate performance of the electrode can be changed by adjusting the crystallographic orientation of the exposed planes, due to the Li^+^-diffusivity difference by a factor of ~10^4^ between [100] and [001] directions in graphite [15,16,17,18]. Therefore, the graphite electrodes having a lower proportion of exposed basal plane are expected to show better rate performance.

**Figure 3:**
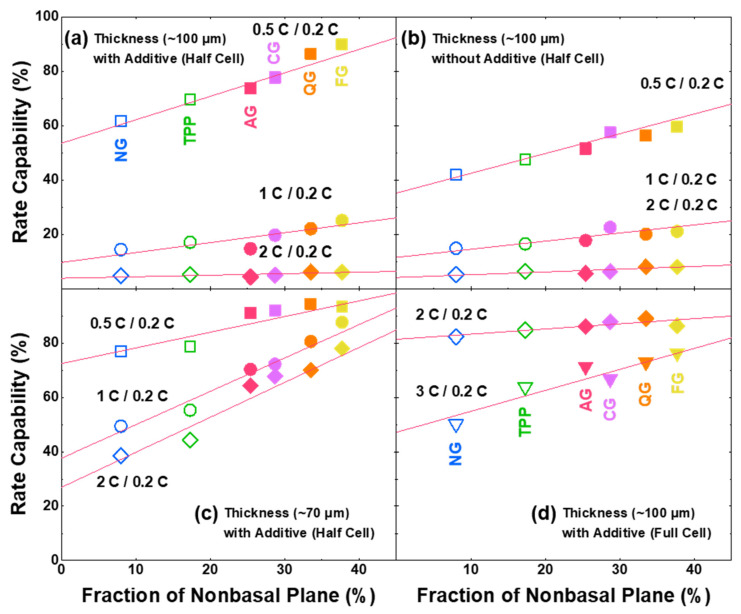
Correlations between fraction of non-basal plane and fast-charging capability with coin cells. Half-cell conditions: (**a**) ~100-μm thick electrode with electrolyte additive, (**b**) ~100-μm thickness without electrolyte additive, and (**c**) ~70-μm thickness with electrolyte additive. (**d**) Full-cell condition: ~100-μm thick graphite electrode with electrolyte additive and LiNi_0.88_Co_0.10_Al_0.02_O_2_ (NCA) cathode.

To confirm the relation of non-basal planes with a fast-charging (lithiation) capability, the capacity values at different C-rates (0.5 C, 1 C, and 2 C compared to 0.2 C) with half cells are plotted with the non-basal-plane fraction (Figure 3a). It can be shown that the fast-charging capability increases linearly with the increased non-basal plane, and capacity decreases with the increasing C-rate, but still exhibits similar linear relationships. Compared to the NG with smaller non-basal planes, the AG shows higher overpotential and more constant-voltage stages in the lithiation profile (Figure S3). In order to confirm that the factors affecting the fast-charging capabilities are more dominant due to the non-basal-plane fraction of the graphite itself than the electrode condition, different cell configurations are tested, depending on the thickness of the electrode with/without additives. Still, the fraction of the non-basal plane affects the fast -charging capability linearly rather than the condition of the electrode (Figure 3a–d). It can be seen that the nanostructural properties of graphite, including lattice constant, local strain, grain size, surface area and Raman analyses have negligible impacts on the fast-charging capability (Figure S4). Although the particle size can affect the diffusion length of Li-ion and the fast-charging eventually, recalling Li-ion only intercalates through non-basal planes ensures that the fraction of the non-basal plane is the more dominant factor. In order to exclude the influence of the Li-metal counter electrode on the observed linearity, the full cell with LiNi_0.88_Co_0.10_Al_0.02_O_2_ (NCA) was tested and the strong correlation of non-basal-plane ratio and fast-charging capability of electrodes was also confirmed in the full cell (Figure 3d). The coefficients of these linear correlations are detailed in Table S2.

**Figure 4:**
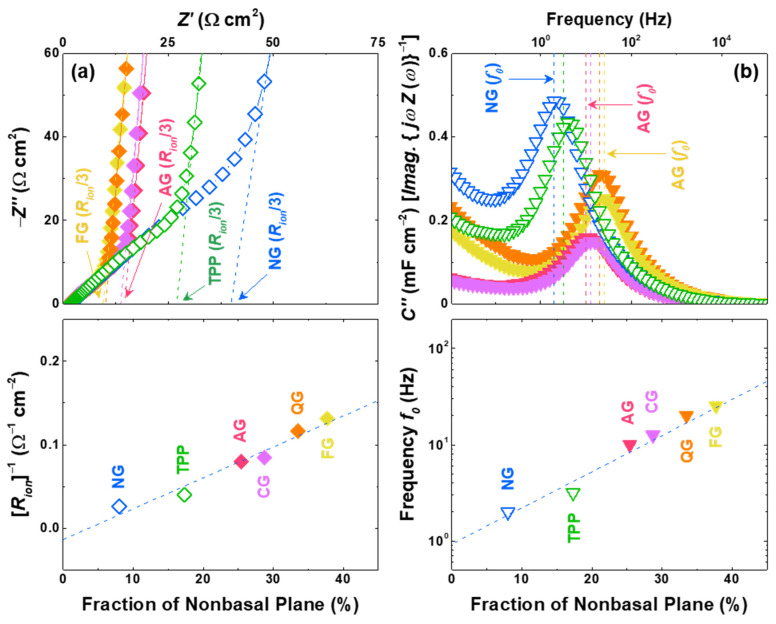
Kinetic properties of graphite symmetric electrodes. (**a**) Impedance spectra at the pristine state (before lithiation) where the intercept at the *x*-axis indicates ionic resistance (*R_ion_*/3) in a porous electrode (top). Dependence of the fraction of non-basal planes on the inverse of the ionic resistance (*R_ion_*)^−1^ (bottom). (**b**) Imaginary part of complex capacitance (top) where the dashed line indicates the frequency for the maximum value (*f*_0_). Dependence of the fraction of non-basal planes on the Li-ion response frequency (*f*_0_) (bottom).

Electrochemical impedance spectroscopy (EIS) are performed with symmetric cells of pristine graphite electrodes to determine the effect of the non-basal plane on the kinetic properties. The ionic resistance (*R_ion_*) values fitted to the transmission line model [45,46,47] are shown as dashed lines, and it can be seen that the *R_ion_* values are smaller for the electrodes with larger non-basal planes (Figure 4a). In order to confirm the correlation between the kinetic properties and non-basal-plane configurations, an alternative electrode parameter, complex capacitances consisting of real (*C′*) and imaginary (*C″*) parts during the non-faradaic process, was derived from the electric double-layer formation model [45,48,49]. As shown in Figure 4b, all of the samples exhibit peak-shaped curves, which implies that the position of the peak frequency in the plot of *C″* equals approximately the time constant reflecting the response time of electric double-layer formation [45,50]. The Li-ion response frequency *f*_0_ also improves, as the fraction of non-basal planes enhances the kinetic properties of the graphite electrode. In addition, the apparent Li-ion diffusivities are measured to support the strong correlation between the non-basal-plane ratio and the kinetic properties of the graphite electrode. Li-ion apparent diffusivities are estimated by both cyclic voltammetry (CV) and EIS, and these values exhibit linear relationships with the increased non-basal plane (Figure S5) [5,9].

### 3.3. Correlation of Pore/Non-Basal Sites to the Cyclability Caused by the Thick SEI Formation

**Figure 5:**
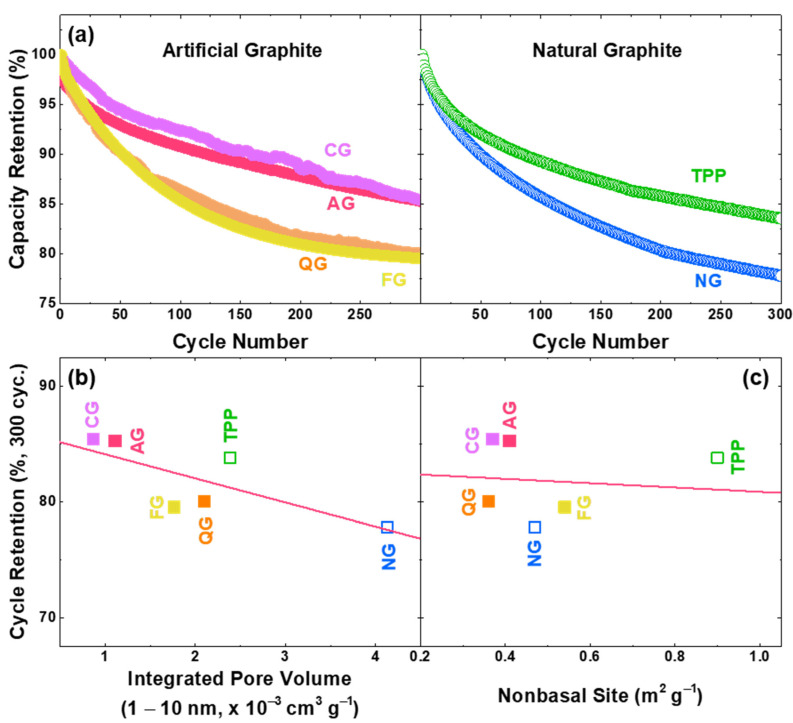
Factors affecting the cycle life of LiNi_0.88_Co_0.10_Al_0.02_O_2_ (NCA) full cells with different graphite anodes. (**a**) Normalized discharge capacity of artificial and natural graphite anodes (potential range of 4.2–2.5 V vs. Li/Li^+^ for 300 cycles with charge/discharge current density of 4.3 mA cm^−2^ (= 1 C)). Dependence of the cycle retention at 300 cycles on the (**b**) pore volume and (**c**) non-basal site. The fitted coefficients for (**b**) and (**c**) are −2.1 (retention%/10^−3^ cm^3^ g^−1^) and −1.9 (retention%/m^2^ g^−1^), respectively.

Since the voltage of electrolyte decomposition occurs in the operation voltage of the graphite, cycle performance can be drastically reduced, as continuous decomposition occurs unless stable SEI is formed on the graphite surface [51,52,53,54,55,56]. Recent studies have reported that irreversible capacity increases linearly with non-basal planes, and the exposed pores in graphite promote SEI formation [22,57,58,59]. In this study, we have additionally examined how non-basal sites affect the cycle life of graphite anodes as well as the pore volume [22]. In order to investigate the correlation between the pore/non-basal sites and cyclability, constant-current charge/discharge cycling tests of these graphites with an NCA cathode (Figure 5) were conducted. The retained specific capacity of full cells at 300 cycles is lower with more pore/non-basal sites. We have also confirmed the cycle life with the pore volume of 1–10 nm in diameter, and the negative linear relationship of the cyclability according to the pore volume was more pronounced than the tendency according to the non-basal site (Figure 5b,c). The BJH method assumes that the pores are cylindrical, but since it is also applied to slit-shaped pores, pores of 1–10 nm appearing in graphite can be considered as defective sites, such as steps or surface roughness [32,33,34]. The pore distributions of graphite samples are shown in Figure S6, and it can be shown that the non-basal sites are not perfectly proportional to the pore volume (Figure S6c). This can be attributed to the volumetric difference of the non-basal constituents, as the edge plane occupies a high portion of non-basal sites, and the nature of defects are more pronounced in the pores with high curvature.

**Figure 6:**
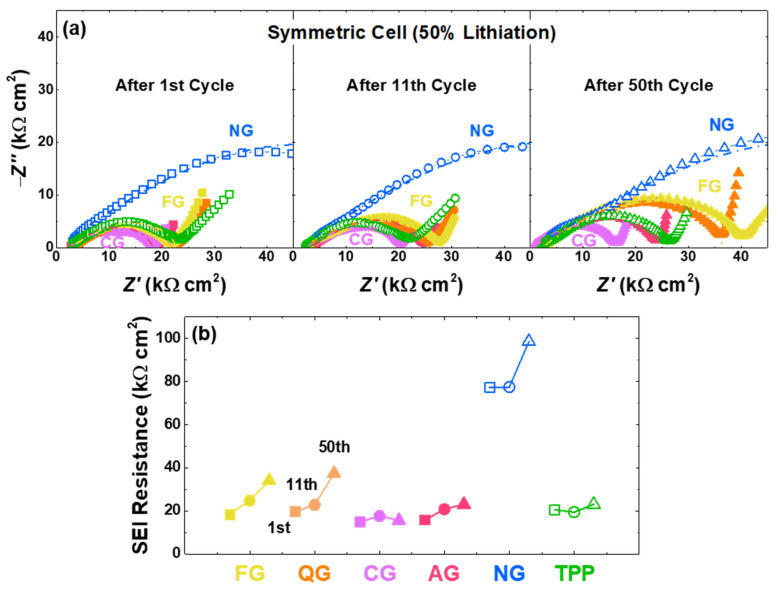
Symmetric-cell electrochemical impedance spectroscopy with various graphite electrodes. (**a**) Impedance spectra of graphite samples at 0.1 V (~50% lithiation). The dashed line predominantly arises from the SEI resistance. (**b**) Changes in the SEI resistance after the 1st, 11th, and 50th cycles of graphite samples.

Impedance analyses using symmetric cells were performed to compare the SEI formation of six different graphites with 50% lithiation after the 1st, 11th, and 50th cycles (Figure 6a,b). The frequencies of semi-circle regions are over 100 Hz, indicating the resistance region attributed to the SEI [22,60,61,62,63]. Although there are differences among the samples, the SEI resistance generally increases as the cycle progresses. For instance, the buildup in the 50th-SEI resistance is significantly larger than the 1st and 11th cycles, indicating the cell degradation. As cycles progress, the degradation accelerates, and the SEI resistance increases as more SEI layers become formed from the previously formed SEI [60,61,62,63]. Such catalyzed degradation looks more pronounced with a higher content of non-basal sites and pores, which means that the SEI formation can occur continuously at the pore and non-basal sites (Figure S7).

## 4. Conclusions

In this work, the electrochemical properties of commercial graphites were categorized with its nanostructural properties and surface heterogeneity of graphite. By careful quantification of the surface heterogeneity, we confirmed that a higher proportion of exposed non-basal (edge and defect) planes is strongly related to the kinetic performance of graphite electrodes. Notably, fast-charging capability has enhanced proportionally to the non-basal sites. On the other hand, the cyclability of the graphite electrode was confirmed to be strongly correlated with the pores. As the impedance analysis on the cycled symmetric cells have exhibited that the increased pore volume have increased the SEI resistance more significantly as the cycles progress, this tendency is valid even when the non-basal site is large. We believe the determination of surface heterogeneity plus pores of graphite materials are powerful parameters that can predict the important electrochemical performances of commercial graphites, and thereby are advantageous for the practical anode design of energy storage materials.

## Data Availability

The data presented in this study are available on request to the corresponding author.

## Supplementary Materials

The following are available online at https://www.mdpi.com/article/10.3390/nano11071813/s1. Table S1: BET surface area, basal plane area, edge plane area, defect site area, and total surface area of graphite samples, derived from the nitrogen adsorption data, Table S2: The correlation between the rate capability and fraction of the non-basal plane, Figure S1: Particle size distribution of graphite samples; Figure S2: Raman spectra of graphite samples and structure parameters obtained through *D*, *G*, and *D’* peaks, Figure S3: Fast-charging capability of graphite samples, Figure S4: Comparison of correlation between fast-charging capability of graphite anodes and other parameters, Figure S5: Kinetic properties of graphite electrodes, Figure S6: Characterization of pores in graphite samples, Figure S7: Comparison of structural parameters from nitrogen adsorption and SEI resistances for six graphite samples.
