# Supplementary material for: Identifying the Association between Surface Heterogeneity and Electrochemical Properties in Graphite
Source: Nanomaterials (Basel). 2021 Jul 13;11(7):1813. doi: 10.3390/nano11071813 (PMC8308424; doi:10.3390/nano11071813)
Supplement: Supplementary file 1 [file nanomaterials-11-01813-s001.zip › nanomaterials-1281678-supplementary.pdf]

Supplementary Material

Identifying the Association between Surface Heterogeneity and Electrochemical Properties in Graphite

Jaewon Kim,[+a,b] Alan Jiwan Yun,[+a]
Kyeu Yoon Sheem,[*b] and Byungwoo Park[*a]

[a]*Department of Materials Science and Engineering, Research Institute of Advanced Materials, Seoul National University, Seoul 08826, Korea*

[b]*Samsung SDI, 130 Samsung-ro, Yeongtong-gu, Suwon, Gyeonggi-do 16678, Korea*

Table S1. BET surface area, basal plane area, edge plane area, defect site area, and total surface area of graphite samples, derived from the nitrogen adsorption data. The characteristics of four artificial-graphite and two natural-graphite powders are as follows: FG (fast charging), QG (fast charging with high specific capacity), CG (cyclability), AG (artificial graphite), NG (natural spherical graphite), and TPP (triphenylphosphine-treated NG).

Sample	FG	QG	CG	AG	NG	TPP
Basal plane (m^2 g^{-1})	0.88 (62.3%)	0.70 (66.5%)	0.94 (71.2%)	1.21 (74.6%)	5.37 (92.0%)	4.30 (82.7%)
Edge plane (m^2 g^{-1})	0.51 (35.9%)	0.31 (29.1%)	0.36 (27.7%)	0.39 (24.3%)	0.16 (3.0%)	0.74 (14.3%)
Defect site (m^2 g^{-1})	0.03 (1.8%)	0.05 (4.4%)	0.01 (1.1%)	0.02 (1.1%)	0.31 (5.0%)	0.16 (3.0%)
Total surface area (m^2 g^{-1})	1.42	1.06	1.31	1.62	5.84	5.20
BET surface area (m^2 g^{-1})	1.37	1.07	1.18	1.27	5.72	4.18

Table S2. The correlation between the rate capability and fraction of nonbasal plane. Correlation coefficients in Figure 3 {the rate capability (%) over the fraction of nonbasal plane (%)} are obtained by linear fitting.

Rate of Charge/Discharge	With Additive. ~100-µm Thickness Half Cell	Without Additive. ~100-µm Thickness Half Cell	With Additive. ~70-µm Thickness Half Cell	With Additive. ~100-µm Thickness Full Cell
**0.5C/0.2C**	0.86	0.73	0.58	-
**1C/0.2C**	0.37	0.30	1.23	-
**2C/0.2C**	0.06	0.10	1.29	0.19
**3C/0.2C**	-	-	-	0.77

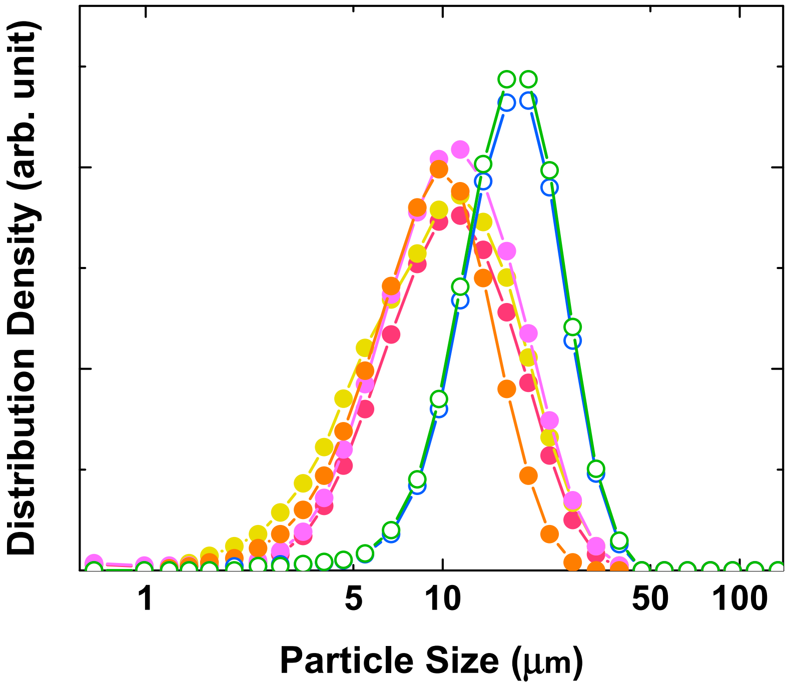

Sample	*D*10 (μm)	*D*50 (μm)	*D*90 (μm)
FG	4.5	9.2	15.8
QG	4.5	9.1	15.8
CG	5.1	10.4	19.7
AG	5.2	10.7	19.9
NG	10.1	17.1	27.3
TPP	9.8	17.0	27.2

Figure S1. Particle size distribution of graphite samples. The parameters of *D*10, *D*50, and *D*90 are related to the volume-based particle size distribution for which 10, 50, and 90%, respectively, of the particles smaller than these sizes.

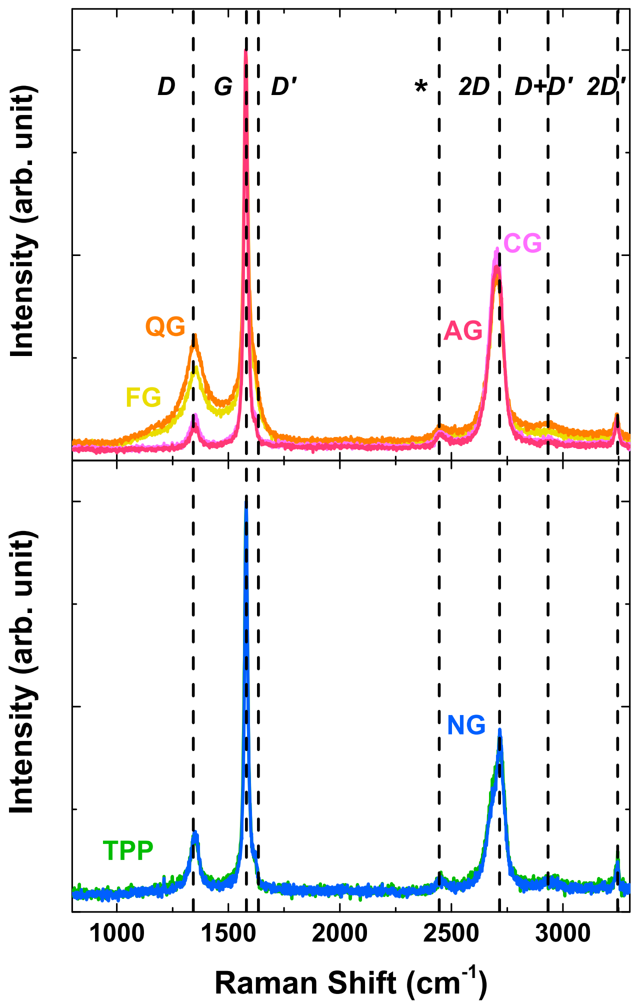

Sample	D/G	D'/G	L_a (nm)	L_D (nm)
FG	0.18	0.10	95.7	26.8
QG	0.26	0.14	65.2	22.1
CG	0.08	0.03	210.0	39.7
AG	0.06	0.03	299.0	47.4
NG	0.15	0.06	111.2	28.9
TPP	0.13	0.04	125.3	30.7

$$\textbf{Crystalite Size } (a-\text{axis}):\; L_a(\text{nm}) = (2.4 \times 10^{-4})\,\lambda_l^4 \left(\frac{I_D}{I_G}\right)$$

$$\textbf{Average distance between defects}:\; L_D(\text{nm}) = \left[(1.8 \times 10^{-9})\,\lambda_l^4 \left(\frac{I_D}{I_G}\right)\right]^{-\frac{1}{2}}$$

λ_l: *excitation laser wavelength* (*nm*)

Figure S2. Raman spectra of graphite samples and structure parameters obtained through *D*, *G*, and *D'* peaks. All the peaks are fitted with Lorentzian functions.

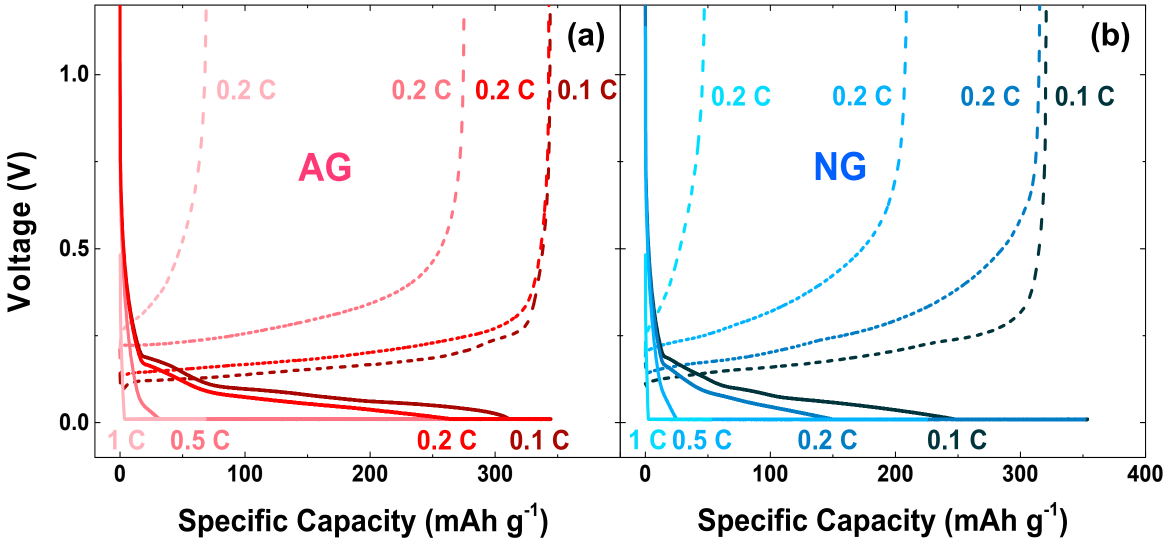

Figure S3. Fast charging capability of graphite samples. Voltage profiles of (a) AG and (b) NG. Lithiation divided into constant-current and constant-voltage (0.01 V) stages, and delithiation with constant current. Solid line indicates lithiation profile (0.1 C, 0.2 C, 0.5 C, and 1 C) and dashed line indicates delithiation profile (0.1 C and 0.2 C).

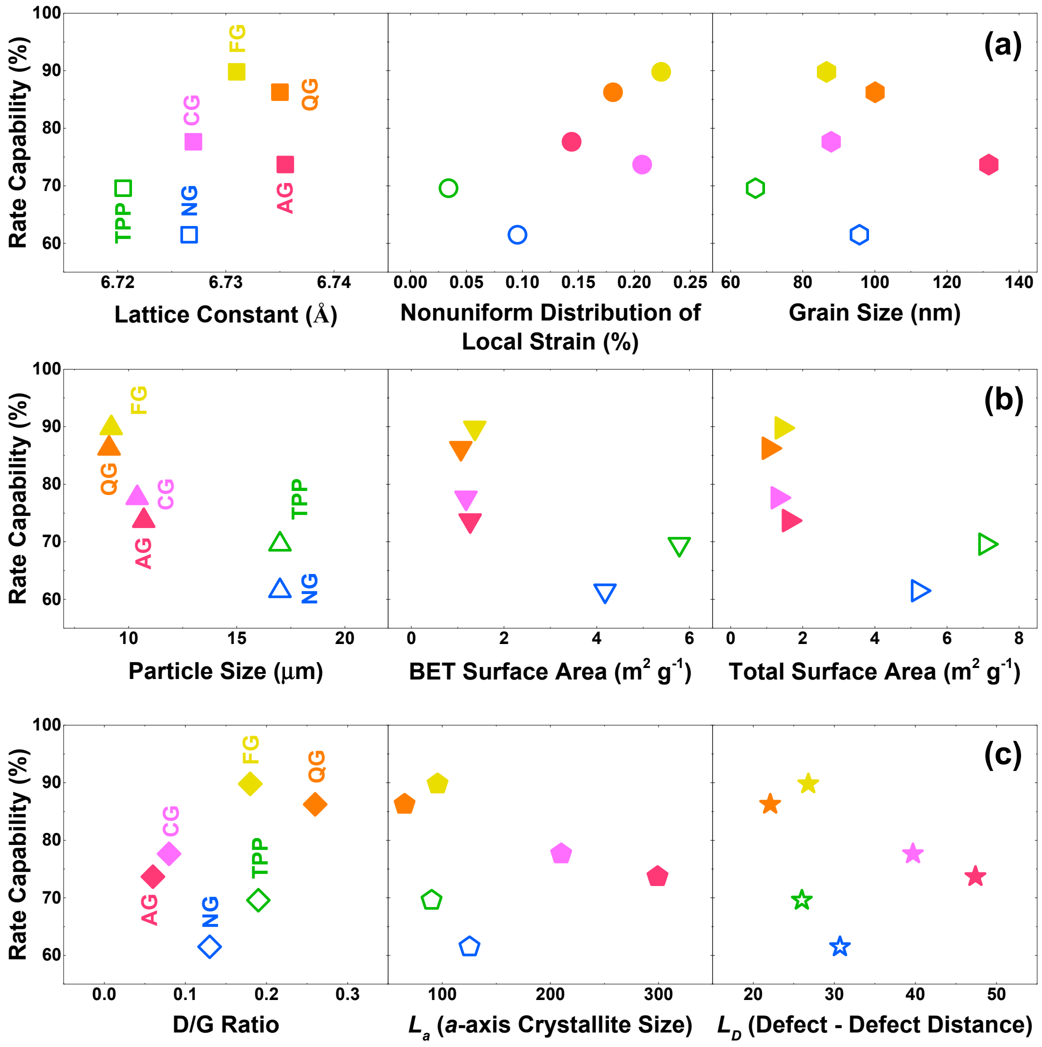

Figure S4. Comparison of correlation between fast charging capability of graphite anodes and other parameters. (a) Structural parameters from the x-ray diffraction analysis. (b) Particle size (*D*50) and surfaces areas (by BET and adsorption-potential distribution) from nitrogen adsorption. (c) Defect parameters from Raman spectroscopy. The charging/discharging was conducted at 0.5C/0.2C.

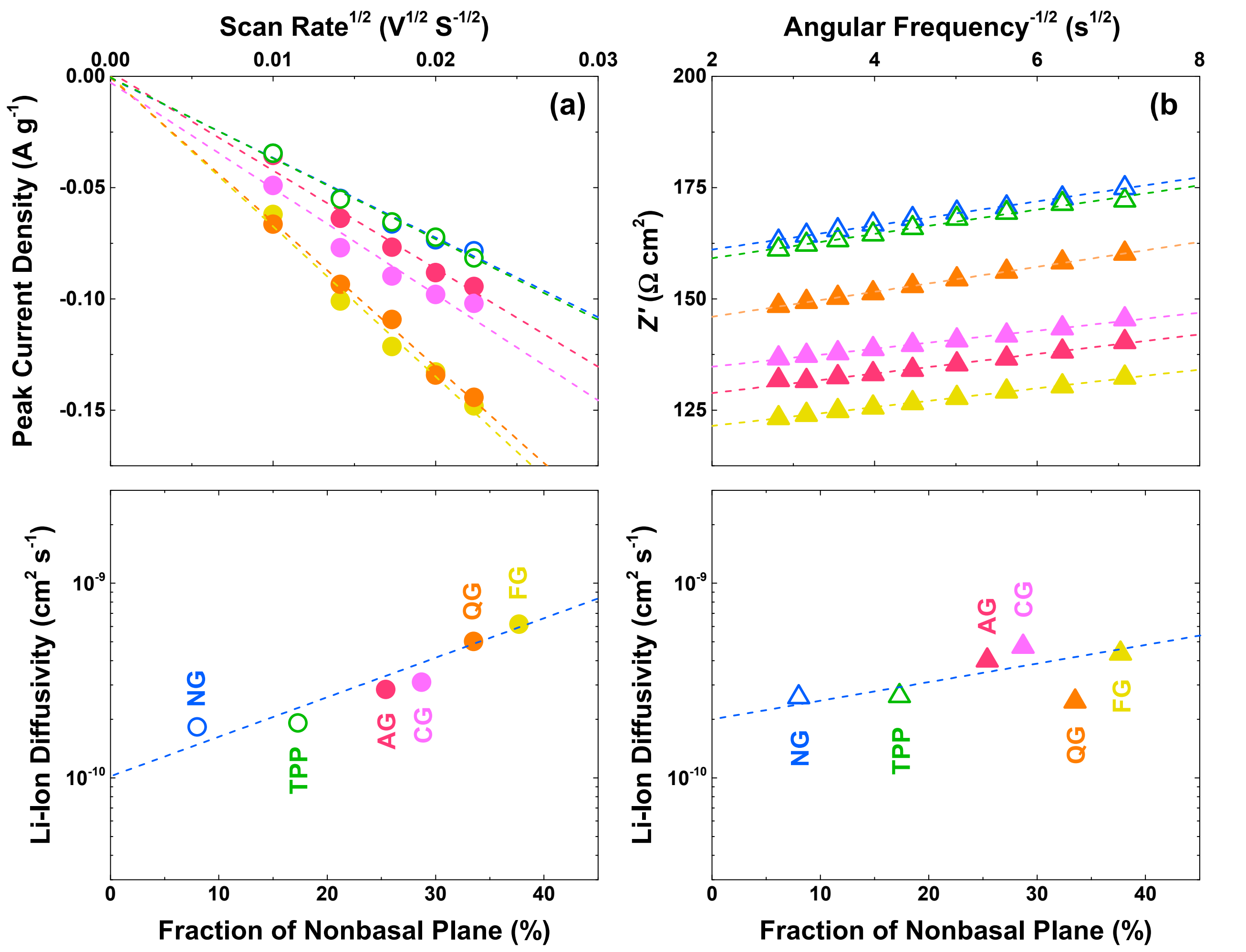

Figure S5. Kinetic properties of graphite electrodes. (a) Relationship between the peak current density (at ~0.1 V) and the square root of the scan rate (top) by cyclic voltammetry, and the corresponding apparent Li^+ diffusivity (bottom). (b) Real part of the complex impedance vs. $\omega^{-1/2}$ (angular frequency) of graphite electrodes at 0.1 V (top) by electrochemical impedance spectroscopy, and the apparent Li^+ diffusivity (bottom).

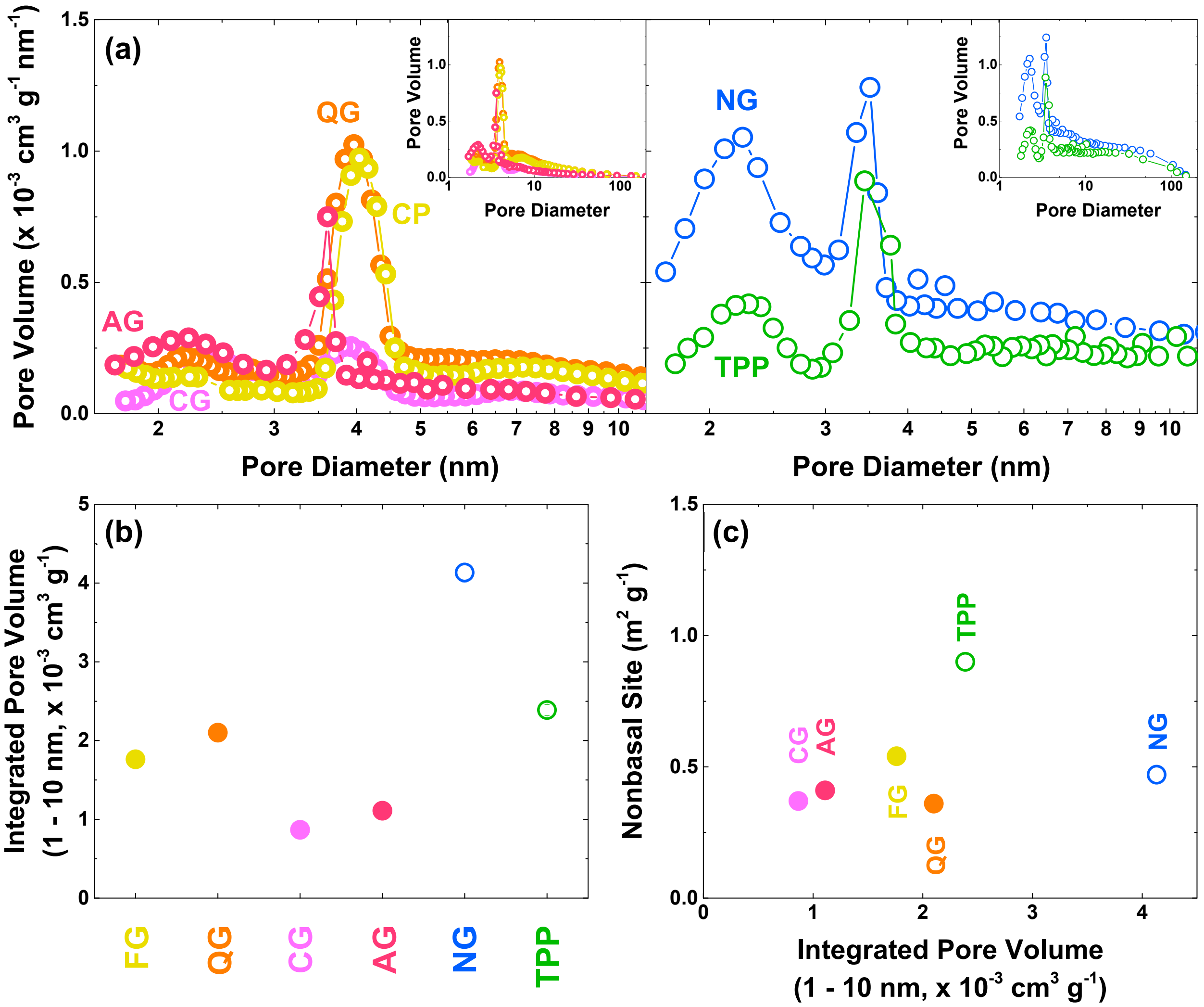

Figure S6. Characterization of pores in graphite samples. (a) Pore distributions (<10 nm) of artificial and natural graphite samples with the total pore distributions in the inset. (b) Integrated pore volume (1 - 10 nm) of the graphite samples. (c) Dependence of the nonbasal site on the integrated pore volume.

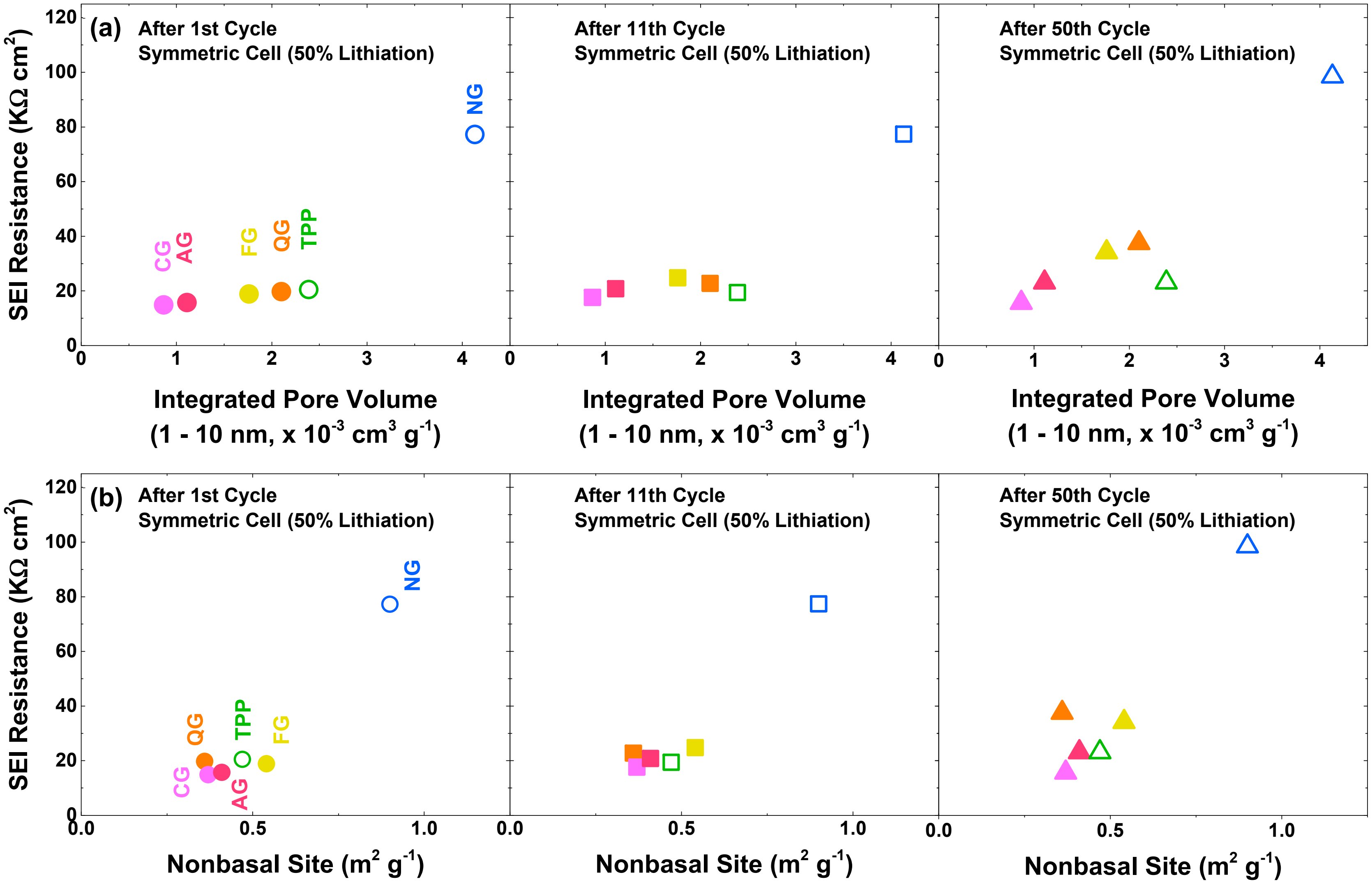

Figure S7. Comparison of structural parameters from nitrogen adsorption and SEI resistances for six graphite samples. Dependence of the SEI resistances after 1, 11, and 50 cycles on the (a) integrated pore volume and (b) nonbasal site.
